# What does Google Trends reveal about the proportion of waterpipe users in the Brazilian population?

**DOI:** 10.1590/S2237-96222023000400004.en

**Published:** 2023-12-18

**Authors:** Fernando Lopes Tavares de Lima, Luís Felipe Leite Martins, André Salem Szklo

**Affiliations:** 1 Instituto Nacional de Câncer, Coordenação de Prevenção e Vigilância, Rio de Janeiro, RJ, Brazil

**Keywords:** Waterpipes, Tobacco, Tobacco Control, Public Health Surveillance, Descriptive Epidemiology, Pipas de Agua, Tabaco, Control del Tabaco, Vigilancia en Salud Pública, Epidemiología Descriptiva, Cachimbos de Água, Tabaco, Controle do Tabagismo, Vigilância em Saúde Pública, Epidemiologia Descritiva

## Abstract

**Objective:**

To analyze the relationship between internet search volume and the prevalence of waterpipe use among young Brazilians in 2019.

**Methods:**

This was a descriptive study with data from Brazil in 2019, using the relative search volume on waterpipes extracted from Google Trends and the proportion of waterpipe users aged between 15 and 24 years, as measured by the National Health Survey (*Pesquisa Nacional de Saúde -* PNS), and aged between 13 and 17 years, as measured by the National Adolescent School-based Health Survey (*Pesquisa Nacional de Saúde do Escolar -* PeNSE). The relationship was assessed by means of Spearman’s correlation.

**Results:**

The point prevalence of waterpipe use across the Brazilian Federative Units (FUs) showed a moderate (r = 0.51; PNS) to strong correlation (r = 0.74 and r = 0.80; PeNSE) with the relative search volume (p-value < 0.05).

**Conclusion:**

Google Trends can support the monitoring system on waterpipe use in the FUs, providing additional information to existing population-based surveys.

## Study contributions

**Main results:**

The search volume for waterpipes by states in Brazil, as measured by Google Trends, showed a moderate to high correlation with the respective proportions of current use and experimentation of waterpipe among the adolescent/young adult population.

**Implications for services:**

The use of Google Trends as a support to the monitoring system for waterpipe consumption in Brazil warrants further exploration in terms of the validity of additional information to existing population surveys.

**Perspectives:**

Expanding the use of Google Trends searches to assess its potential in monitoring other health risk products and detecting (or predicting) incidence or seasonality of health-related events.

## RESUMEN

**Objetivo:**

Analizar la relación entre el volumen de búsquedas en Internet y la prevalencia del uso de narguile entre los jóvenes brasileños en 2019.

**Métodos:**

Estudio descriptivo con datos de Brasil en 2019, utilizando el volumen relativo de búsqueda sobre narguile extraído de Google Trends y la proporción de usuarios de narguile entre 15 y 24 años, medida por la Encuesta Nacional de Salud (PNS), y con edades entre 13 y 17 años, medida por la Encuesta Nacional de Salud Escolar (PeNSE). La relación fue respaldada por el método de correlación de Spearman.

**Resultados:**

La prevalencia puntual del uso de narguile en las Unidades Federativas brasileñas presentó una correlación moderada (r = 0,51; PNS) a fuerte (r = 0,74 y r = 0,80; PeNSE) con el volumen relativo de búsqueda (p-valor < 0,05).

**Conclusión:**

Google Trends puede apoyar el sistema de monitoreo del uso de narguile en las UFs, ofreciendo información adicional a las encuestas poblacionales existentes.

## RESUMO

**Objetivo:**

Analisar a relação entre o volume de pesquisas na internet e a prevalência de uso de narguilé entre jovens brasileiros em 2019.

**Métodos:**

Estudo descritivo com dados do Brasil em 2019, utilizando o volume relativo de pesquisa sobre o narguilé extraído do Google Trends e a proporção de usuários de narguilé com idade entre 15 e 24 anos, medida pela Pesquisa Nacional de Saúde (PNS), e com idade entre 13 e 17 anos, medida pela Pesquisa Nacional de Saúde do Escolar (PeNSE). A relação foi avaliada por meio da correlação de Spearman.

**Resultados:**

A prevalência pontual de uso de narguilé das Unidades da Federação (UFs) brasileiras apresentou correlação moderada (r = 0,51; PNS) a forte (r = 0,74 e r = 0,80; PeNSE) com o volume de pesquisas relativo (p-valor < 0,05).

**Conclusão:**

O Google Trends pode apoiar o sistema de monitoramento sobre o uso do narguilé nas UFs brasileiras, oferecendo informações adicionais às pesquisas populacionais existentes.

## INTRODUCTION

Tobacco epidemic causes 161,000 deaths annually in Brazil and accounts for BRL 125 billion in direct and indirect costs.^1^ Among tobacco-derived products, waterpipes stand out due to their significant appeal to young people in terms of flavor/aroma additives, social bonding and the belief that they are less harmful to health.^2-4^ In fact, the proportion of waterpipe users among individuals aged 18-24 quadrupled between 2013 and 2019 (0.6% *versus* 2.4%).^5^

Furthermore, approximately 27% of Brazilian adolescents aged 13 to 17 have already tried this product.^6^ This allure is channeled by the tobacco industry, which interferes with the implementation of the National Tobacco Control Policy (*Política Nacional de Controle do Tabaco -* PNCT) to ensure that additives can still be used in waterpipes, while also promoting its marketing, often through illegal means, among “new nicotine consumers”.^7,8^

Thus, the quest for information about this product emerges as a strategic topic to be explored by infodemiology. This refers to the science of distribution and determinants of information in electronic media, specifically on the internet, with the aim of fostering public policies.^9^ In this context, Google Trends is the most commonly used tool for identifying population interests in health information,^9,10^ given that, based on a subject identifier term, it can extract its search volume and compare it across specific locations and/or periods. As the results are displayed in real-time, it stands as a potential analysis tool for public health decision-making, including the field of cancer prevention and control.^11^ The aim of this article was, therefore, to analyze the relationship between internet search volume and the prevalence of waterpipe use among young Brazilians in 2019.

## METHODS

A descriptive study was conducted based on internet search volumes available on Google Trends^10,11^ and prevalence data on waterpipe use from the National Health Survey (*Pesquisa Nacional de Saúde* - PNS)^4^ and the National Adolescent School-based Health Survey (*Pesquisa Nacional de Saúde do Escolar* - PeNSE).^5^

The analysis was restricted to the year 2019, as it had the most recent national data and was the first year in which data on the prevalence of waterpipe use were collected in PeNSE. Data on 124,811 young people from PeNSE and 10,460 from PNS were used. Details about the sampling plans and data collection for the PNS and PeNSE can be found in specific publications.^5,6^ The following variables were used:

1) Relative search volume: The relative search volume on the internet is automatically normalized by the Google Trends tool (https://trends.Google.com/) for a sample of searches in a specific location and period, ranging from zero (when there is no interest) to 100 (peak interest).^10,11^ The absolute number of searches used to calculate the relative search volume is not provided.^12^ Data extraction was performed in May 2023 in the “interest by sub-region” chart, using the subject “waterpipe”, limited to Brazil in 2019, and encompassing “all categories” of “Web search”.
2) Prevalence of ever-use of waterpipe and in the last 30 days: It was obtained from data collected by PeNSE. All students aged 13 years and older were asked: *Have you ever tried waterpipe in your lifetime?* and *In the last 30 days, which of these other tobacco products have you used?*, with waterpipe as one of the answer options.
3) Prevalence of current waterpipe use: It was obtained from data collected by the PNS. All selected individuals aged 15 years and older were asked: *Do you currently smoke any tobacco products?*; and if so, *On average, how many times do you use waterpipe to smoke per day or per week currently?* Current users were defined as those who reported some regular usage frequency, even if less than once a month.

The analyses were limited to individuals aged 13 to 17 years (PeNSE) and 15 to 24 years (PNS). This choice is justified by the fact that around 95% of Brazilian smokers start smoking before the age of 25 years^5^ and that approximately 80% of waterpipe users are between 15 and 24 years old.^4^

The correlation between the relative search volume and the prevalence of waterpipe use was obtained using non-parametric Spearman’s test. This test was chosen after verifying, through the use of a histogram and the Shapiro-Wilk test that the variables did not show normal distributions. Furthermore, to help visualize the relationship between the two variables, Locally Weighted Scatterplot Smoothing (LOWESS) was used, which is a non-parametric strategy for fitting a smooth curve to data points. The analyses were performed using Python on the Google Colab platform.

Google Trends data are publicly available and their use does not require approval of a Research Ethics Committee. The National Research Ethics Committee approved the PNS (Opinion No. 3,529,376 on 8/29/2019) and PeNSE (Opinion No. 3,249,268 on 8/4/2019).

## RESULTS

**Figure 1:**
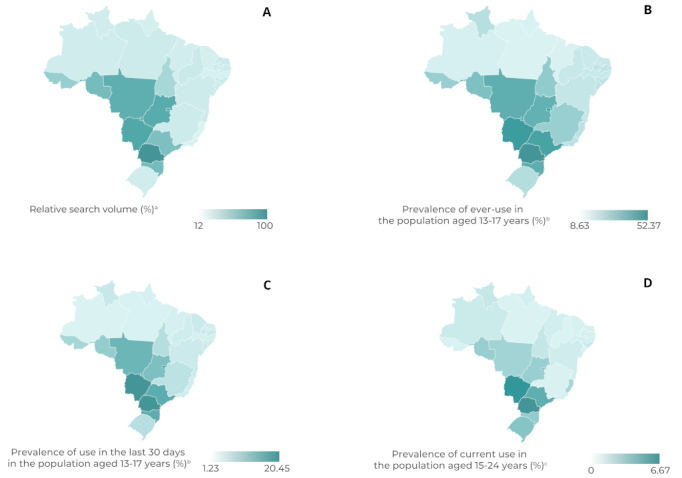
Relative search volume (A) and point prevalence of ever-use of waterpipe (B), in the last 30 days (C) and currently (D), by Federative Units, Brazil, 2019

The relative search volume of Google Trends and the point prevalence of waterpipe use obtained from PeNSE and PNS were higher in the states of the South region (with the exception of the state of Rio Grande do Sul) and the Midwest region when compared to the other Federative Units (FUs) (with the exception of the state of São Paulo). For each FU, the point prevalence of ever-use, among adolescents aged 13 to 17 years, was higher than the respective prevalence of use in the last 30 days, which, in turn, was higher than the point prevalence of current use among those aged 15 to 24 years (Figure 1 and Table 1).

a) Google Trends; b) National Adolescent School-based Health Survey (Pesquisa Nacional de Saúde do Escolar - PeNSE); c) National Health Survey (Pesquisa Nacional de Saúde - PNS).

**Table 1:** Relative search volume and point prevalence of waterpipe use in the Federative Units, Brazil, 2019

Federative Unit	Relative search volume^a^ (%)	Prevalence of ever-use: 13-17 years old^b^ (%)	Prevalence of use in the last 30 days: 13-17 years^b^ (%)	Prevalence of current use: 15-24 years old^c^ (%)
Acre	46.00	24.80	6.95	0.05
Alagoas	20.00	15.69	2.88	0.72
Amapá	15.00	8.98	1.66	0.20
Amazonas	17.00	9.61	1.23	0.56
Bahia	18.00	14.19	2.79	0.37
Ceará	15.00	12.88	3.02	0.09
Distrito Federal	79.00	50.59	18.90	3.57
Espírito Santo	12.00	16.30	2.38	1.99
Goiás	78.00	38.91	11.50	2.51
Maranhão	16.00	8.69	1.66	0.24
Mato Grosso	75.00	39.84	13.48	2.16
Mato Grosso do Sul	82.00	48.91	20.45	6.52
Minas Gerais	18.00	25.07	4.53	0.02
Pará	18.00	8.63	1.41	- ^d^
Paraíba	18.00	15.40	3.03	0.22
Paraná	100.00	52.37	20.36	6.67
Pernambuco	14.00	13.24	1.87	0.21
Piauí	20.00	13.45	2.41	- ^d^
Rio de Janeiro	12.00	16.17	2.34	0.22
Rio Grande do Norte	19.00	13.13	2.05	0.09
Rio Grande do Sul	17.00	18.27	5.16	3.24
Rondônia	63.00	31.90	8.94	2.55
Roraima	17.00	17.54	3.16	0.77
Santa Catarina	64.00	40.62	15.23	2.81
São Paulo	59.00	45.90	15.34	4.77
Sergipe	18.00	15.65	3.23	- ^d^
Tocantins	38.00	26.86	5.38	0.87

a) Google Trends; b) National Adolescent School-based Health Survey (Pesquisa Nacional de Saúde do Escolar - PeNSE); c) National Health Survey (Pesquisa Nacional de Saúde - PNS); d) In the selected sample, no current waterpipe users were identified, which does not necessarily imply the absence of waterpipe use in the FU.

**Figure 2:**
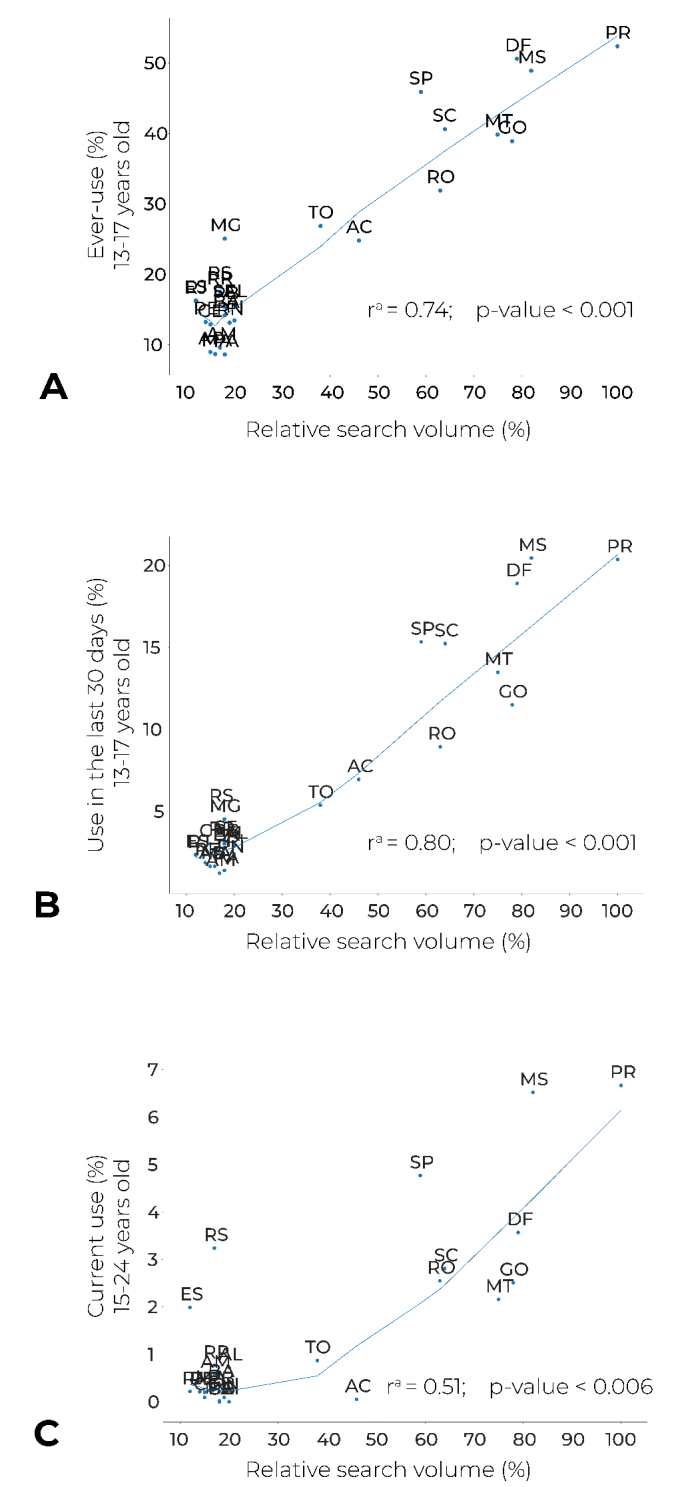
Correlation between relative search volume and point prevalence of ever-use of waterpipe (A), in the last 30 days (B) and currently (C), by Federative Units, Brazil, 2019

The point prevalence of ever-use of waterpipe and in the last 30 days among adolescents aged 13 to 17 years, obtained from PeNSE, showed strong and statistically significant correlations with the relative search volume (r = 0.74 and r = 0.80, respectively; p-values < 0.001) (Figure 2A and 2B). On the other hand, the point prevalence of current waterpipe use among individuals aged 15 to 24 years obtained from the PNS showed a moderate and statistically significant correlation (r = 0.51; p-value = 0.006) (Figure 2C).

a) Spearman’s correlation.

## DISCUSSION

The volume of searches for information on waterpipes in the Brazilian FUs showed a high correlation with the respective proportions of current use (or experimentation) by the adolescent/young adult population of the country.

Brazil has a comprehensive system for monitoring the tobacco epidemic. It comprises a series of questions about smoking behavior included in periodic national surveys conducted in both the young and adult populations,^5,6^ in addition to data on the production and importation of tobacco-derived products.^13^ This enables the assessment of the effectiveness of the various actions aimed at reducing the initiation and/or promoting cessation of nicotine-containing products.^8^ This system is essential for evaluating the ongoing impact of the tobacco industry’s interference with the PNCT, aiming to slow down the reduction in the proportion of tobacco product users in the country.^7^ In this sense, the present study suggests that Google Trends warrants further exploration for its potential to complement the monitoring of this epidemic. It can capture, without having to wait for the periodic epidemiological surveys and subsequent dissemination of their results, the constant changes in the tobacco industry’s *modus operandi*.^7,14^

Google Trends has been used in other countries^15-17^ to measure changes in user behavior resulting from legislative, economic and/or educational measures aimed at combating tobacco-derived products or as a response to the industry’s marketing strategies in reaction to such measures.^15,17^ These changes can be expressed both through searches for information related to cessation^15-17^ and by substitution for another product that allows for the maintenance of nicotine dependence, especially electronic smoking devices.^18^

Furthermore, Google Trends has been used to understand how unforeseen acute events, such as the COVID-19 epidemic, can trigger changes in smokers’ behavior.^19^ In Brazil, it has been used to predict the incidence and outbreaks of infectious diseases^19-21^ and to analyze the impact of cancer prevention and control campaigns.^22,23^ In other words, beyond the issue of smoking, Google Trends has the potential to swiftly assist the Brazilian National Health System in planning and evaluating public actions and policies. Its advantages include free and real-time availability, Google’s dominance in the search engine market, the representation of actual behavior, user anonymity and the offer of data on sensitive topics.^12^

The limitations of this study include the analysis being restricted to 2019, the focus on Google search engine users, the lack of information on the sample design and its slight variation, depending on the day the survey is conducted^10^, and the influence of unknown factors on internet behavior.^12^

The results suggest that, despite its limitations, the use of Google Trends deserves further exploration as support to the traditional and well-stablished monitoring system on waterpipe use for assessing the effectiveness of actions aimed at combating smoking in the country.
